# Perspectives in Aptasensor-Based Portable Detection for Biotoxins

**DOI:** 10.3390/molecules29204891

**Published:** 2024-10-15

**Authors:** Congying Li, Ziyuan Zhu, Jiahong Yao, Zhe Chen, Yishun Huang

**Affiliations:** 1 College of Environment and Public Health, Xiamen Huaxia University, Xiamen 361024, China; 2 School of Forensic Medicine, Shanxi Medical University, Jinzhong 030600, China; 3 Key Laboratory of Forensic Toxicology of Ministry of Public Security, Jinzhong 030600, China; 4 China Institute for Radiation Protection, Taiyuan 030000, China; 5 Institute of Analytical Technology and Smart Instruments, Xiamen Huaxia University, Xiamen 361024, China

**Keywords:** biotoxin, aptamer, portable detection, lateral flow assay, personal glucose meters, smartphone

## Abstract

Biotoxins are pervasive in food and the environment, posing significant risk to human health. The most effective strategy to mitigate the risk arising from biotoxin exposure is through their specific and sensitive detection. Aptasensors have emerged as pivotal tools, leveraging aptamers as biorecognition elements to transduce the specificity of aptamer-target interactions into quantifiable signals for analytical applications, thereby facilitating the meticulous detection of biotoxins. When integrated with readily portable devices such as lateral flow assays (LFAs), personal glucose meters (PGMs), smartphones, and various meters measuring parameters like pH and pressure, aptasensors have significantly advanced the field of biotoxin monitoring. These commercially available devices enable precise, in situ, and real-time analysis, offering great potential for portable biotoxin detection in food and environmental matrices. This review highlights the recent progress in biotoxin monitoring using portable aptasensors, discussing both their potential applications and the challenges encountered. By addressing these impediments, we anticipate that a portable aptasensor-based detection system will open new avenues in biotoxin monitoring in the future.

## 1. Introduction

The occurrence of biotoxins in food safety and environmental pollution is a primary global problem of increasing concern. Biotoxins, also referred to as natural toxins, are typically non-proteinaceous small molecules, peptides, or proteins synthesized by various living organisms, including plants, microbes, and animals [1]. They can be categorized into four primary groups based on their origin: animal toxins (snake venom), phytotoxins (abrin and ricin), microbial toxins (bacteria toxins and mycotoxins), and marine toxins (fish venom and shellfish toxins) [2]. Detailed information on these common biotoxins, including their source, toxic effects, and associated symptoms, is provided in Table 1. Biotoxins can exert detrimental effects on the nervous system, potentially leading to partial damage or impairment of nerves and neurons, resulting in symptoms such as dyspnea, respiratory failure, and, in severe cases, death. Moreover, they can also target and inflict damage on kidney and liver tissues, inducing acute and chronic toxicity [3]. Notably, these toxins can persist in water, soil, and food samples even after the demise of the organisms that produced them, thereby contributing to environmental contamination and ecological imbalance [4]. Consequently, developing on-site and real-time biotoxin monitoring strategies is imperative to safeguard environmental stability and human health.

Chromatographic and spectrographic methodologies, such as high-performance liquid chromatography (HPLC), liquid chromatogram tandem mass spectrometry (LC-MS/MS), thin-layer chromatography (TLC) [5], and capillary electrophoresis [6], represent two traditional and widely used strategies for biotoxins detection. These techniques afford precise, sensitive, and concurrent detection of biotoxins in multi-component samples, making them widely adopted in laboratory settings. However, these methods are encumbered by significant drawbacks, including the necessity for expensive instrumentation, cumbersome sample preparation processes, and the requirement for skilled operators. These inherent limitations make these approaches unsuitable for on-site monitoring of biotoxins in agriculture sectors, food industries, and environmental screening.

Recently, the emergence of point-of-care testing (POCT) has led to a transformative shift in biotoxin detection, facilitating rapid identification and immediate intervention through the employment of portable biosensors. This innovation extends the scope of testing beyond traditional laboratory settings to include environments such as schools, nursing homes, pharmacies, and even home environments, utilizing miniaturized diagnostic devices that are readily available in physical stores or e-commerce platforms [7]. Devices like lateral flow assays (LFAs), personal glucose meters (PGMs), smartphones, pH meters, pressure meters, thermometers, pregnancy test strips, and microfluidic chips are widely distributed in markets [8]. These tools allow for the rapid analysis of various health indicators; for instance, PGM can analyze blood sugar levels within minutes, while pregnancy can be confirmed by the test strip with just drops of urine [9]. Likewise, portable biosensors that utilize POCT technologies can detect biotoxin contamination in water or food with ease. These portable biosensors meet essential requirements, including portability, affordability, ease of use, and high sensitivity, addressing the urgent need for instantaneous detection [10]. Consequently, they hold immense potential for curbing the proliferation and transmission of biotoxins, averting critical health crises, and reducing the economic burden associated with biotoxin management, thereby playing a pivotal role in safeguarding human health and environmental stability.

Both antibodies and aptamers could serve as biorecognition elements in the construction of portable biosensors for biotin detection. However, antibodies are limited due to their limited efficacy in distinguishing structurally similar targets in a complex and heterogeneous food or environmental matrix. Aptamers, small single-stranded oligonucleotides, offer advantages over antibodies such as greater selectivity and affinity, cost-effectiveness, faster production, and strong performance under inhospitable conditions. Aptasensors, utilizing aptamers as recognition molecules, have become the preferred choice for monitoring harmful substances with high affinity and specificity, such as pesticides, antibiotics, pollutants, insecticides, allergies, poisons, and so on [11]. They are extensively applied to different biotoxins detection and have been known to overcome the interference of impurities successfully [12]. Several reviews have focused on aptasensors for the detection of biotoxins. Zhao et al. [13] scrutinized developments from 2016 to 2021 in aptasensors targeting shellfish toxins, discussing the underlying principles, signal transduction technologies, and response types. Bilibana et al. [14] surveyed aptasensors functionalized hybrid nanomaterials, designed to monitor and mitigate algal toxins in aquatic environments. Shkembi et al. [15] highlighted the utilization of optical and electrochemical methods in constructing aptasensors to counter mycotoxins, emphasizing advancements in material utilization and immobilization strategies to enhance sensitivity and selectivity. Zahraee et al. [16] explored diverse approaches employing aptamers as biorecognition elements in biosensors for phycotoxin detection, covering colorimetric, fluorescent, electrochemical, and microfluidic-based aptasensors. These reviews have demonstrated that aptasensors exhibited significant potential in enhancing food safety through the precise, rapid, and cost-effective detection of biotoxins. However, to the best of our knowledge, none of the aforementioned reviews have exclusively addressed the latest advancements in portable aptasensors incorporating POCT technologies for biotoxin detection. The miniaturized devices incorporating aptasensors provide a broad market value in a variety of research areas.

This is the first comprehensive analysis of recent research advancements in portable aptasensors for biotoxin detection, utilizing technologies such as LFAs, PGMs, smartphones, pH meters, pressure meters, thermometers, pregnancy test strips, and microfluidic chips. This review aims to bridge the existing gap in the literature by providing a meticulous analysis and summary of the merits, constraints, and challenges posed by the strategies. Additionally, our focus is on providing a comprehensive review of the current scenario of portable aptasensors designed for biotoxin detection, along with potential future prospects and pathways for development. These portable aptasensors hold great promise in diverse research fields, including but not limited to food safety, environmental science, and analytical science.

**Table 1:** Four types of common biotoxins, sources, toxic effects, and symptoms.

Types	Representative	Source	Toxic Effect	Symptoms	Ref.
Animal toxins	Bungarotoxin (α-BGT and β-BGT)	*Bungarus multicinctus*	Nervous system, lung and heart	Wound pain, localized swelling, salivation, nausea, vomiting, coma, respiratory failure and death	[17]
	Cardiotoxins (CTXs)	*Naja atra*	Nervous system, heart	Damage to the heart, arrhythmias, myocardial hypoxia	[18]
Plant toxins	Abrin	*Abrus Precatorirus*	Liver, kidneys, spleen, blood cells, lung and heart	Diarrhea, vomiting, colic, tachycardia, kidney failure, respiratory failure and death	[19]
	Ricin	*Ricinus communis*	Liver, kidney, cardiovascular and respiratory center	Nausea, vomiting, abdominal pain, diarrhea, convulsions, shock and death	
Microbial toxins	Aflatoxin (B1, B2, G1, G2, M1, M2)	*Aspergillus*	liver, kidney, gastrointestinal tract	Fever, vomiting, anorexia, jaundice, followed by ascites, swelling of the lower extremities and rapid death	[20]
	Ochrotoxin A (OTA)	*Aspergillus* and *Penicillium*	Kidney, liver, immune system	Mental depression, loss of appetite, weight loss, digestive disorders, kidney damage, and often abortion	[21]
	Zearalenone (ZEN)	*Fusarium* and *Gibberella*	Reproductive system, nervous system, heart, kidney, liver and lung	Nausea, chills, headache, depression, ataxia, miscarriage, stillbirth and teratology	[22]
	Botulinum toxin	*Clostridium botulinum*	Nervous system	Ptosis, diplopia, strabismus, dysphagia, dizziness, muscle weakness, dyspnea and death	[23]
	Staphylococcal enterotoxins	*Staphylococcus aureus*	Immune system	Nausea, vomiting and diarrhea	[24]
Marine toxins	Saxitoxin (STX)	*Alexandrium catenella, Gonyaulax* *catenella*, and *Alexandrium tamarense*	Nervous system, cardiovascular system	Nausea, vomiting, diarrhea, local skin tingling sensation, neuromuscular paralysis, arrhythmia, death	[25]
	Okadaic acid (OA)	*Dinophysis* and *Prorocentrum*	Intestines tract, liver and nerves	Bellyache, queasiness, chilli-ness, abdominal pain, Cancer	[26]
	Ciguatoxins	*Gambierdiscus toxicus*, *Prorocentrum* and *Pyrocystis*	Nervous system and respiratory center	Weakness of the limbs, inability to speak, drooping eyelids, staggering, coma, respiratory failure and death	[27]
	Tetrodotoxins	*Gambierdiscums toxincus*, puffer fish	Nervous system and sodium ion channel inhibitors	Respiratory failure and death	[28]
	Palytoxins (PTX)	*Palythoa* and *Zoanthus*	Cardiovascular, nervous system, digestive tract and kidney	Blisters, bleeding or ulcers, dyspnea, vomiting, extensive gastrointestinal bleeding, shock, and death	[29]

## 2. Aptamers Targeting Biotoxins

### 2.1. Selection Principle of Aptamers

Aptamers are short oligonucleotides, typically ranging from 20 to 80 nucleotides in length, that are screened in vitro from random libraries for their ability to bind to specific targets with high selectivity and affinity [30]. The isolation of aptamers was first characterized independently in 1990. Tuerk and Gold introduced the in vitro selection process termed SELEX (systematic evolution of ligands by exponential enrichment) [31]. Subsequently, the term ’aptamer’ from aptus, meaning ’fitting’ in Latin, was created [32]. The SELEX procedure encompasses a series of stages, including incubation, binding, elution, amplification, and conditioning [33]. The target is first incubated with a random library consisting of nucleotide sequences (20~80 nt). Following this, non-binding sequences are eluted, retaining only those sequences that exhibit target-specific binding. The retained sequences are then amplified by PCR before the cycle is repeated to further enhance affinity through successive enrichment. The procedure finishes in sequencing to explore key characteristics of aptamers, including their molecular structure, affinity, specificity, and selectivity for the target [34,35]. The traditional SELEX process has undergone numerous enhancements by streamlining selection cycles and increasing efficiency.

Bead-based SELEX (Bead SELEX) is a widely used technique for SELEX, wherein target molecules are immobilized on a solid support, such as agarose, sepharose, or sephadex beads. Various affinity tags and resins, including histidine (His), biotin, maltose-binding protein (MBP), glutathione S-transferase (GST), nickel-nitrilotriacetic acid (Ni-NTA), glutathione, and amylose, make this approach versatile and suitable for a broad range of target molecules [36,37]. With the targets pre-immobilized on the beads, they are then available for incubation with oligonucleotide libraries, allowing specific aptamers to capture the targets through affinity interactions.

Magnetic bead-SELEX (Mag-SELEX) utilizing magnetic microbeads to immobilize the target was established in 1997 [38]. In this method, the target is immobilized on magnetic beads (MB) by coupling or “biotin-streptavidin” interactions. The aptamer library is then incubated with the MB-target complexes, which can be facilely separated from unbound sequences by a magnetic separator [39]. As a widely used technique, Mag-SELEX is simple to handle and does not involve expensive devices. Recently, several biotoxins immobilization-improved Mag-SELEX have been reported [40,41,42,43,44].

However, targets immobilized on the beads or on the magnetic beads can potentially raise two key concerns: insufficient exposure of target binding sites and potential natural conformational changes of targets. These issues may reduce SELEX efficiency or even result in SELEX failure. To address these challenges, capture-SELEX has been developed, wherein the oligonucleotide library is immobilized on a carrier containing complementary sequences instead of the target immobilization. Specific target binding to the nucleotide sequence triggers its detachment from the carrier, followed by amplification and additional selection cycles until optimal binding activity is reached [45].

Capillary electrophoresis-SELEX (CE-SELEX) is another modified method to perform selection in free solution, utilizing capillary electrophoresis in the separation step. This approach distinguishes bound sequences from the random library based on differential migration times, which is a consequence of the varying charges and sizes of the aptamer-target binding complexes compared to unbound sequences. CE-SELEX not only necessitates fewer screening rounds (typically 2–4 rounds), but also significantly enhances selection efficiency [46]. CE-SELEX was initially employed for screening large targets, which presented certain limitations. However, Yang et al. introduced a novel approach by successfully applying CE-SELEX to a small-molecule target. Although the change in mobility of the complex compared to unbound sequences was minimal, PCR amplification enabled sufficient enrichment, even with the collection of only a small portion of the complex. This work significantly enhanced the applicability of CE-SELEX for the screening of small-molecule targets [47]. Numerous subsequent studies have confirmed that CE-SELEX is a convenient, rapid, and high-affinity method for aptamer screening. It is now widely applied in the selection of aptamers for small molecules.

Graphene oxide SELEX (GO-SELEX) is a non-immobilized method to overcome the requirement of immobilizing target or library on the surface. Here, the library is incubated with the target before exposure to GO sheets. DNA not engaged in target binding adheres to GO through π-π stacking, allowing for the separation of binding sequences via centrifugation. The binding sequences are amplified and subjected to subsequential enrichment to achieve the targeted aptamers [48].

### 2.2. Overview of Aptamers Targeting Biotoxins

Until now, aptamers have been selected for a wide range of targets. This burgeoning field has matured considerably, with a wealth of publications describing the deployment of aptamers as biorecognition elements in biosensors for biotoxin detection, a trajectory that has been on an exponential rise. Aptamers confer a myriad of unparalleled advantages over antibodies when utilized as bioreceptors in biosensors, as summarized in Table 2. Compared to antibodies, aptamers (single-stranded DNA or RNA) are inherently more flexible and significantly smaller, with attributes that are highly beneficial in biosensor applications. Compared to antibodies, aptamers (single-stranded DNA or RNA) are inherently more flexible and significantly smaller, with attributes that are highly beneficial in biosensor applications. Firstly, smaller aptamers exhibit minimal batch-to-batch variation and lower production costs due to their relatively short sequences, which can be synthesized chemically. Secondly, aptamers have low immunogenicity and can target small analytes that are often inaccessible to antibodies. Thirdly, their smaller size allows for precise chemical modifications to incorporate various reporting tags, including fluorescent [49], electrochemical [50], and enzymatic [51] tags for signal reporting. Additionally, aptamers possess higher structural stability under harsh conditions and adopt appropriate conformations for interacting with specific targets. Moreover, aptamers are also much less susceptible to denaturation and degradation at a range of ionic conditions, pH, temperatures, and other storage conditions, thereby promising longer shelf lives. Once identified and sequenced, they can be artificially synthesized, eliminating the need for repetitive screening processes. Their inherent properties provide significant advantages in the development of biotoxin-specific biosensors. Owing to their high homogeneity with DNA, the workflows for the design and optimization of biotoxin biosensors are well streamlined.

Our investigation indicates that aptamers have taken their place as important biorecognition elements, revolutionizing the way researchers detect and quantify biotoxins. A detailed summary of various prevalent biotoxin aptamers is presented in Table 3, encompassing aspects such as the target, aptamer sequence, selection method, type of aptasensor, linear range, and the limit of detection (LOD). Furthermore, we have analyzed the secondary structures of each aptamer, a crucial factor given that the unique recognition abilities of aptamers are grounded in their highly adaptive folding capabilities.

Mag-SELEX stands as the predominant selection method for biotoxin-targeting aptamers, which utilizes magnetic nanoparticles to immobilize biotoxins and facilitate swift magnetic separation. As a paradigm, Guo et al. [40] successfully screened and characterized aptamer specific to α-conotoxin MI (CTX-MI), achieving an affinity constant (Kd) of approximately 0.524 µM. Frohnmeyer et al. [41] utilized a robotic magnetic separator to enhance the Mag-SELEX process, thereby mitigating contamination risks and reducing manual intervention. This approach yielded aptamers with high affinity for cholera toxin (CT), one of which exhibited a Kd value of 48.5 nM and was subsequently employed in crafting an enzyme-linked aptamer assay. Furthermore, some scientists have focused on different selection methods that can simplify the screening process but preserve high efficiency for biotoxins. The utilization of GO-SELEX offers significant advantages for the development of aptamers to small molecules compared to the conventional selection methods. Gao et al. [52] were pioneers in successfully selecting an aptamer targeting Gonyautoxin1/4 (GTX), achieving high affinity and specificity. Zhang et al. [53] utilized Capture-SELEX to pinpoint aptamers effective against gymnodimine-A (GYM-A), thereby laying the groundwork for a novel aptasensor that incorporates biolayer interferometry (BLI) technology. This aptasensor demonstrated a linear detection range from 28 to 444 μg·kg^−1^ and a limit of detection (LOD) as low as 3.15 μg·kg^−1^.

The binding domain of an aptamer can sometimes be identified through methods such as truncation, mutation, and chemical probing. In many instances, successful truncation of an aptamer yields a shorter sequence that retains the target-binding affinity of the original molecule. Moreover, under certain circumstances, truncations and mutations can even enhance selectivity and affinity by eliminating unwanted nucleotides that interfere with target binding. The truncated aptamers can reduce the cost of synthesis and make aptamers easier to incorporate into various sensor platforms. For example, Li et al. [42] demonstrated that the terminal-fixed aptamer had a binding affinity 145 times greater than its predecessor. Based on this, the crafted aptasensor exhibited a low detection limit of 0.04255 μg·kg^−1^ and achieved high recoveries ranging from 98.21% to 114.1% in analyzing STX of seawater samples, suggesting high applicability of both the modified aptamer and the developed aptasensor. Yang et al. [43] ingeniously designed an anti-Aflatoxin B1 (AFB1) aptamer through a rational approach involving truncation and splicing. This aptamer exhibited 188 times higher affinity compared with the original. Building on this, a colorimetric assay-based aptasensor was developed for AFB1 detection, showcasing impressive accuracy and high recovery rates, thereby affirming the efficacy of the developed aptasensor.

## 3. Portable Aptasensors for Biotoxin Determination

Traditionally, biotoxin detection has relied heavily on centralized, sophisticated analytical instrumentation. These bulky instrumentations are extremely expensive and require specialized personnel for handling, along with involving redundant sample preparations and clearance processes, rendering them inappropriate for on-site analysis. Recent cumulative advancements in biosensor technology have facilitated the development of portable biosensors that incorporate POCT, demonstrating significant potential for real-time biotoxin monitoring. These portable aptasensors stand out for their ultra-low detection limits and reduced size compared to traditional benchtop setups. Beyond their sensitivity and specificity, they cater to essential requirements such as user-friendliness for novices, affordability, and ease of use, thereby facilitating swift actions and remedies. They stand as a desirable platform for sensitive, on-site biotoxin assays in agricultural fields, food and beverage industries, and environmental screenings.

In this section, we have presented a variety of portable aptasensors available for biotoxin detection, including those based on LFAs, PGMs, smartphones, and other portable devices. We aim to provide a comprehensive discussion on the performance and potential of these aptasensors in the contemporary landscape.

### 3.1. Portable Aptasensors Based on LFAs

**Figure 1:**
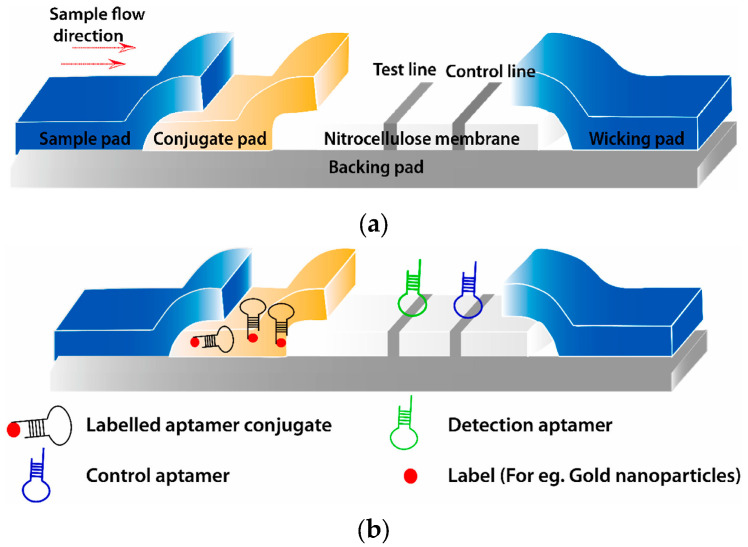
(**a**) Fundamental assemblies required for the construction of LFA; (**b**) Schematic diagram illustrating aptamer-based LFA. Reprinted with permission from Ref. [63]. Copyright 2023, Elsevier.

LFA is a widely recognized test strip-based assay, typically in the form of immunocompetition or aptamer-target capture. A standard lateral flow assay (LFA) comprises four fundamental components: a sample pad, a conjugate pad, a detection pad, and a wicking pad. Included among these, the detection pad predominantly features two lines: a control line (C line) and a test line (T line), as illustrated in Figure 1a. Compared to traditional ELISA and other immunological techniques, LFA stands out in biotoxin monitoring in the field of biotoxins monitoring due to its operational simplicity, rapidity, portability, quantitative detection capabilities, and potential for multiplexed analysis. To setup the aptasensor-based LFA, as depicted in Figure 1b, a labeled aptamer conjugate is firstly positioned in the conjugate pad. The detection aptamer is placed on the T line, which must exhibit high specificity and sensitivity to accurately detect the target, even within a mixed sample. Meanwhile, the control aptamer, designed to be continuously complementary to the labeled aptamer conjugate, is situated on the C line [63]. This ensures the display of a distinct red band, irrespective of the target’s presence or absence, serving as a validation of the strip’s functionality. Portable aptasensors integrated LFA technology exhibit remarkable sensitivity, stability, portability, simplicity, cost-effectiveness, and user-friendliness. Typically, these qualitative assays are signaling by colorimetric and fluorescent methodology.

#### 3.1.1. Colorimetric LFA Aptasensors

Colorimetric LFA aptasensors have been described as a potent technique for the detection of biotoxins, predicated on color alteration. They are engineered to straightforwardly signify the occurrence or the estimated concentration of biotoxins via discernible discoloration or color intensity on the T line. In contrast to other approaches, the detected results can be acquired directly through the naked eye, strip reader, or digital reading, demonstrating the appealing features of easy manipulation, swift reaction, cost-effectiveness, and no need for specialized personnel. Currently, colorimetric LFA aptasensors have been the most commonly and simplest instruments in the field for a rapid response of biotoxins.

Within the realm of colorimetric LFA aptasensors, gold nanoparticles (GNPs) are predominantly utilized as labels, as depicted in Figure 1b. Surpassing other labels, GNPs, as spherical particles, are amenable to facile functionalization, exhibit augmented stability, possess a higher affinity towards biomolecules, and deliver superior optical signaling [64]. Zhou et al. [65] employed a competitive-based LFA aptasensor for the visual detection of OTA using GNPs, wherein the OTA and the immobilized complementary DNA (cDNA) in the T line bind competitively to the mobile GNPs-aptamer conjugate. Without OTA, the probe engages with the GNPs-aptamer conjugate on the T line, culminating in a visible line. In the presence of OTA, the target competed with the DNA probe, resulting in the absence of a visual line at the T line. This assay demonstrated a detection threshold of 1 μg·kg^−1^, exhibiting negligible cross-reactivity and circumventing the utilization of specialized instruments. Likewise, aptamer-functionalized GNPs have been employed for the monitoring of MC-LR and AFB1 via the competitive model, achieving the LOD of 2.5 μg·kg^−1^ and 0.1 μg·kg^−1^, respectively [66,67].

The use of nanozymes as the signal labels in colorimetric LFA aptasensors is becoming increasingly appealing due to their dual functionality: they not only exhibit their own intrinsic color, but also catalyze the oxidation of chromogenic substrates, thereby enabling signal amplification. Zhu et al. [68] reported on a CuCo@PDA nanozyme-based LFA aptasensor designed for advanced, sensitive, and on-site monitoring of AFB1. The black CuCo@PDA was conjugated with a specific aptamer, generating a clear visual colorimetric signal on the T line through competitive sensing, owing to its rich amino functional groups and outstanding peroxidase-like activity. Additionally, it catalyzed the coloration of the TMB-H_2_O_2_ system for signal amplification, enabling a visual and dual-readout detection of AFB1. The amplified signal, observed visually, demonstrated an accurate and ultrasensitive detection of AFB1 with the lower limit of detection (LOD) of 2.2 × 10^−3^ μg·kg^−1^ and a desirable recovery in the range of 95.11–113.77%.

#### 3.1.2. Fluorescent LFA Aptasensors

Fluorescent LFA aptasensors exhibit a broad range of response, tailorability, and high sensitivity compared to the colorimetric LFA and thus have great potential to become an ideal quantitative method for biotoxin detection. However, the sensitivity predicated on fluorescent molecules may be attenuated due to photobleaching alongside chemical and metabolic degradation. Accordingly, it is essential to use suitable fluorescent materials, such as organic fluorescent material, quantum dots (QDs), and upconversion nanoparticles (UCNPs), to enhance the analytical properties of LFA aptasensors.

**Figure 2:**
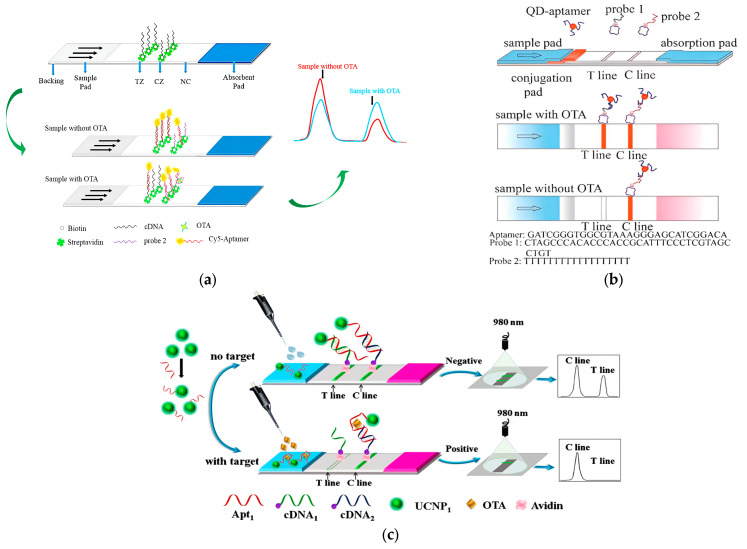
(**a**) Working principle of Cy5-labeled LFA aptasensor for detection of OTA. Reprinted with permission from Ref. [69]. Copyright 2018, MDPI. (**b**) Schematic representation of fluorescent LFA aptasensor based on QD for OTA analysis. Reprinted with permission from Ref. [73]. Copyright 2011, Royal Society of Chemistry. (**c**) Mechanism diagram of UCNP-labeled LFA aptasensor for OTA detection. Reprinted with permission from Ref. [78]. Copyright 2018, Springer Nature.

The organic fluorescent material Cy5 belongs to the water-soluble 3H-indocyanine type bio-fluorescent labeling dye, which is easy to couple with aptamers and has a good fluorescence intensity and is thus commonly used in the development of fluorescent LFA aptasensors. Zhang et al. [69] presented the LFA aptasensor predicated on the competitive reaction between Cy5-labeled aptamer and cDNA at the T line, revealing an inverse proportionality between the OTA concentration in samples and the fluorescence intensity ratio (Figure 2a). Without OTA, the Cy5-labeled aptamer is predominantly captured via cDNA, engendering pronounced fluorescence intensities at two lines. Conversely, the presence of OTA facilitates its binding to aptamer, diminishing the quantity of aptamer hybridized to cDNA on the T line and thereby attenuating fluorescence intensities. This assay exhibited an exemplary linear relationship within the range of 1~1000 μg·kg^−1^ and a LOD of 0.40 μg·kg^−1^. Analogously, an LFA aptasensor was devised for the detection of Type-B aflatoxins employing Cy5-labeled aptamer, wherein the sample containing Type-B aflatoxins and immobilized cDNA vie for binding to the Cy5-labeled aptamer. This assay discerned concentrations as low as 0.16 μg·kg^−1^ within a linear range of 0.2 to 20 μg·kg^−1^ [70]. A parallel endeavor by Zhu et al. [71] entailed the design of a novel dual competition encompassing target–aptamer–antigen and target–aptamer–cDNA integrated with the LFA, aimed at detecting AFB1 with a LOD of 0.1 μg·kg^−1^ and a sensitivity range of 0.1–1000 μg·kg^−1^. These findings underscore the superior sensitivity and specificity of Cy5-labeled LFA aptasensors in biotoxin analysis. Nonetheless, organic dyes bear the drawbacks of broad emission ranges, narrow absorption ranges, and photobleaching. The advent of functionalized nanoparticles proffering fluorescence signals, such as QDs and UCNPs, has garnered burgeoning attention in aptasensor advancements. These nanoparticles, serving as both acceptor and donor fluorophores, ameliorate the aforementioned issues, thereby widening the scope of fluorescent LFA aptasensor technology.

QDs are semiconducting nanoparticles endowed with remarkable fluorescence properties, exhibiting robust photoluminescence (PL) upon ultraviolet (UV) light excitation. This characteristic positions QDs as a viable alternative to organic fluorescent molecules, making them ideal fluorescent labels [72]. Unlike organic fluorescent molecules, QDs exhibit a higher resistance to metabolic degradation, augmented stability, and an elevated molar absorption coefficient. Wang et al. [73] described a simple fluorescent LFA aptasensor based on QDs, targeting OTA as a model toxin (Figure 2b). The aptasensor demonstrated a LOD of 1.9 μg·kg^−1^ with a rapid monitoring time of merely 10 min. The versatility of QDs extends to multiplexed detection owing to their customizable sizes and compositions. Subsequent endeavors have harnessed QDs in fluorescent LFA for multiplexed detection. Bruno et al. [74] reported a fluorescent LFA aptasensor for multiplexed assays targeting intimin protein, lipopolysaccharide (LPS), and Shiga toxin 1 (Stx 1), aiming to improve the monitoring of a broad range of pathogenic *Escherichia coli* (*E. coli*) through surface intimin proteins and secreted Stx1 using aptamers and quantum dots (QDs). The integration of LFA with multiplexed detection resulted in enhanced sensitivity, reduced cross-reactivity, and shorter assay times, achieving detection limits as low as 100 *E. coli* bacterial cells and 10 ng of Stx 1. However, the employment of QDs is encumbered by their high cost and the requirement for external apparatus, such as UV lamps, for quantification.

UCNPs are inorganic crystalline nanomaterials that are capable of emitting Near Infrared Ray (NIR) light (typically 980 nm) into visible and ultraviolet emission light. Due to their unique optical properties, UCNPs can be excited with NIR light to reduce autofluorescence background interference and allow for deeper transmission into analytes owing to minimized light scattering. This results in highly sensitive detection [75,76,77]. Wu et al. [78] harnessed UCNPs to architect a fluorescent LFA based on the competitive interaction between OTA and its cDNA for a UCNP-labeled aptamer (Figure 2c). In the absence of OTA, the UCNP exhibited robust properties, rendering two discernible green fluorescence signals on both the C and T lines under 980 nm laser excitation. Conversely, with OTA, a single green fluorescence signal was displayed on the C line. Spanning a range of 5 to 100 μg·kg^−1^, the relative fluorescence intensity exhibited a direct proportionality to the OTA concentration, with efficacious detection achievable within 15 min. Jin et al. [79] constructed a multiplexed LFA employing carboxylated UCNP to generate multi-colored signals, enabling the concurrent detection of three analytes (mercury ions, OTA, and *Salmonella*) upon excitation with 980 nm laser radiation. Utilizing three distinct color channels, aptamers specific to each analyte were labeled with UCNPs to yield fluorescence signals devoid of specific binding and crossover reactions. Each target was assayed on a separate T line, as UCNPs of three colors were tethered to detection aptamers, thereby expediting and economizing the monitoring process. Additionally, a paper disc UCNP-based LFA was conceived for multiplex detection targeting *Salmonella*, OTA, MC-LR, Hg^2+^, and Pb^2+^, exhibiting high sensitivity and specificity [80]. This innovative system amalgamated multi-channels into a singular connected disc, obviating crossover reactions and augmenting detection throughput. Within a 30-min span, quintuple targets were detected, manifesting the system as a rapid and highly sensitive apparatus. Nonetheless, the requisite of a near-infrared laser for UCNP excitation necessitates integration with a compact reading device to facilitate point-of-care biotoxin monitoring.

### 3.2. Portable Aptasensors Based on PGMs

PGMs are widely utilized due to their handiness (i.e., ‘pocket’ size), cost-effectiveness, ease of use, and reliable quantitative results. However, their application has traditionally been limited to glucose detection, restricting their use to non-glucose analytes. To overcome this limitation, researchers have devised methods to convert non-glucose analyte signals into glucose signals via signal identification and transformation mechanisms. This innovation significantly broadens the applicability of PGMs for point-of-care detection of various non-glucose targets [81]. Many studies combine PGM-based biosensors with various signal amplification mechanisms to enhance monitoring efficiency. The most prevalent amplification mechanisms involve enzymatic reactions and the use of advanced nanomaterials as nanocarriers. This section summarizes the notable work by the analytical principles, considers the challenges of aptasensor-based PGMs in detecting biotoxins, and explores the potential advancements through the incorporation of signal amplification technologies.

#### 3.2.1. Enzymatic Reactions

**Figure 3:**
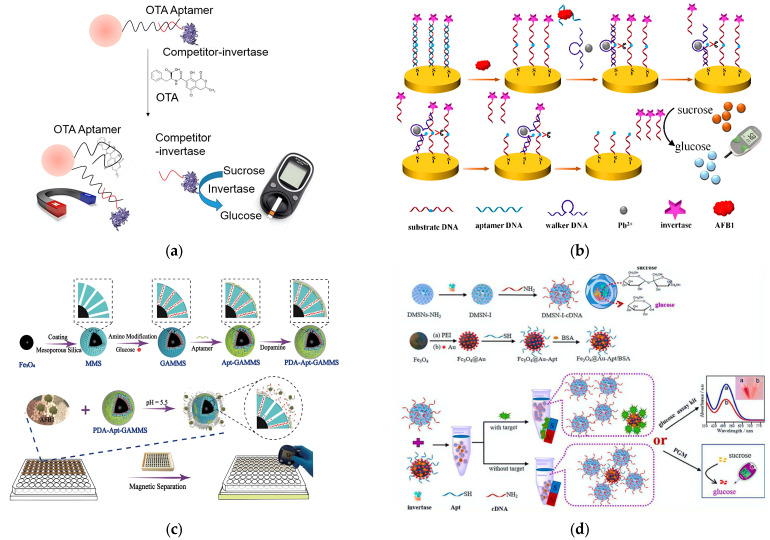
(**a**) Schematic illustration of PGM-based aptasensor for detecting OTA via a structure-switching aptamer. Reprinted with permission from Ref. [82]. Copyright 2016, Royal Society of Chemistry. (**b**) Schematic representation of PGM-based aptasensor for AFB1 analysis by DNA walking machine. Reprinted with permission from Ref. [84]. Copyright 2018, American Chemical Society. (**c**) Mechanism diagram of PGM-based aptasensor for AFB1 detection using the “dual gates” locked nanodevice. Reprinted with permission from Ref. [86]. Copyright 2019, American Chemical Society. (**d**) Schematic diagram of aptasensor based on DMSNs-I nanoreactors with PGM as readout for detecting AFB1. Reprinted with permission from Ref. [87]. Copyright 2021, Elsevier.

A classic and typical approach to reuse a PGM-based biosensor for non-glucose targets involves the addition of a specific enzyme that transforms sucrose into glucose, which can then be detected by the PGM. The effective applications of DNA-functionalized invertase have demonstrated the good suitability between invertase and nucleic acid amplification techniques. Gu et al. [82] established an aptasensor based on PGM for the quantitative determination of OTA (Figure 3a). The aptamer was hybridized with cDNA modified with invertase to form double-stranded (dsDNA). With OTA, the aptamer/OTA recognition triggered the release of cDNA-invertase, with the invertase catalyzing the hydrolysis of sucrose to glucose. The portable PGM successfully monitored OTA, achieving a detection limit of 3.66 μg·kg^−1^. This supported the applicability of portable aptasensors based on PGM for biotoxin analysis. Similarly, Qiu et al. [83] utilized alkynyl DNA-invertase comprising ten bases to conjugate with aptamer, forming a short dsDNA. Owing to the poor stability of the short-hybridized dsDNA, they adopted Cu to the ‘click’ ligation generated between aptamer and DNA-invertase, forming covalent bonding and thus greatly reducing the background signal. With OTA, the aptamer, being occupied by OTA, resulted in the deconjugation of the short-hybridized dsDNA, preventing the ‘click’ ligation between the aptamer and DNA-invertase. Consequently, most of the DNA-invertase strands were released, resulting in a high PGM signal. This setup displayed a high sensitivity for OTA monitoring, achieving a detection limit of up to 0.072 μg·kg^−1^.

In addition to employing DNA-functionalized invertase, the catalytic capability of DNAzyme to facilitate DNA cleavage has also been successfully harnessed. Yang et al. [84] introduced a portable aptasensor based on PGM for detecting AFB1 by using the concept of DNA walking machine. In this assay, the aptamer was hybridized with substrate DNA on the electrode (Figure 3b). AFB1 prompted the aptamer to disassociate from the electrode, enabling walking DNA (DNAzyme) to cleave the substrate DNA in the presence of Pb^2+^, subsequently releasing the invertase, hydrolyzing sucrose to glucose, which was detected via the PGM. Exploiting the capacity of catalytic cleavage by DNAzyme, a high sensitivity for AFB1 monitoring was achieved, with a linear range from 0.0062 to 3.1 μg·kg^−1^ and a LOD of 0.0031 μg·kg^−1^. Furthermore, Zhang et al. [85] devised an aptasensor based on magnetic beads and a DNAzyme for signal amplification to detect OTA in red wine. This setup exhibited high sensitivity with a detection range spanning five orders of magnitude and a LOD of up to 0.88 × 10^−3^ μg·kg^−1^. The aptamer initially conjugated to DNAzyme, thereby inhibiting substrate cleavage activity. Upon the introduction of OTA, a specific interaction occurred between OTA and the aptamer, facilitating the displacement of the DNAzyme. Subsequently, the DNAzyme associated with the substrate DNA anchored on the magnetic beads, instigating a cascade of hydrolysis reactions. This process enabled the liberation of invertase into the supernatant, culminating in the generation of a significant quantity of glucose. As a result, the successful transduction of OTA into a quantifiable glucose measurement was achieved utilizing the PGM.

In summary, sensing methodologies combining enzyme reactions with PGMs have been developed for biotoxin detection. However, these approaches without sophisticated designs often exhibit low detection sensitivity. Moreover, enzymatic reactions are prone to interference under certain circumstances, like varying pH and temperature levels. Therefore, integrating advanced nanomaterials as nanocarriers alongside signal amplification techniques can effectively mitigate these challenges.

#### 3.2.2. Advanced Nanomaterials as Nanocarriers

It is noteworthy that advanced nanomaterials, when utilized as glucose-encapsulated nanocarriers, exhibit excellent biocompatibility. This characteristic helps in averting many interferences with enzymatic structure and function, thereby preserving the enzyme activity to a significant extent. Additionally, these nanocarriers simplify the process by reducing the need for washing and separation steps through a straightforward trigger mechanism. In particular, mesoporous silica nanoparticles (MSNs) have garnered attention as nanocarriers due to their large pore volumes, extensive available inner surface areas, and ease of functionalization. For instance, Wang et al. [86] reported a novel and portable method for monitoring AFB1. This method employed a target-responsive nanodevice using a polydopamine aptamer combined with aminated magnetic mesoporous silica nanocomposites (PDA-Apt-GAMMS) for glucose loading with a PGM readout (Figure 3c). In the absence of AFB1, glucose is more securely gated into the pores of PDA-Apt-GAMMS, which results in a low background signal. When AFB1 is present, the aptamer-AFB1 binding reaction triggers the escape of glucose from the pores, which can be detected by a PGM. This proposed aptasensor successfully achieved precise recognition and then sensitive detection of AFB1, achieving low detection limits of 0.02 μg·kg^−1^ and a dynamic response range of 0.03 to 8 μg·kg^−1^. Furthermore, this sensing strategy can be easily adapted for monitoring other biotoxin analytes in real samples by merely modifying the corresponding sequence of the aptamer.

In addition to encapsulating glucose, MSNs can also encase enzymes within nanoscale confining spaces, thereby preserving the enzymes’ catalytic activity. Yang et al. [87] designed dendritic mesoporous silica nanoparticles (DMSNs) nanoreactors by covalently immobilizing invertase within the inner areas and modifying cDNA on the surface. This design facilitated signal amplification through a substantial enzyme load amount and high bioactivity recovery (Figure 3d). When the targets (AFB1) were absent, Fe_3_O_4_@Au-aptamers captured DMSN nanoreactors due to the hybridization reaction between cDNA and aptamers. This configuration could efficiently catalyze the hydrolysis of sucrose to glucose, which could then be quantitatively detected by PGM. Upon the addition of AFB1, a competition ensued between AFB1 and DMSN nanoreactors for Fe_3_O_4_@Au-aptamers. This competition resulted in the release of invertase and a subsequent decrease in catalytic products. Compared to other reported single enzyme-labeled aptasensors, the sensing strategy employing DMSN nanoreactors as signal labels demonstrated a wider linear range, better stability, higher sensitivity, and a lower detection limit.

Utilizing advanced nanomaterials as nanocarriers for signal amplification has been well integrated into the sensing platform owing to their exceptional loading capability and the preservation of enzyme activity to the maximum extent. Although the developed strategy showcases the advantages of signal amplification, there remains a lack of precise control over the pore size and volume of the nanomaterials, necessitating more standardized methods through validated processes. Furthermore, it is worth exploring other functionalized nanomaterials to enhance detection sensitivity.

### 3.3. Portable Aptasensors Based on Smartphones

Aptasensors based on smartphones have largely replaced expensive detection equipment and time-consuming monitoring techniques, thereby receiving wide research attention. Smartphones, with their ability to interface with various hardware and software, exhibit significant potential, transforming into sensors, system controllers, and data processors [88]. Based on the mode of signal transduction, aptasensors can be categorized into optical and electrochemical models, with the latter presumably being more prevalent [89,90]. In the early stage, Zahra et al. discussed the principles of designing optical and electrochemical aptasensors [91,92]. Smartphones can be seamlessly integrated with these aptasensors for a wide range of applications encompassing colorimetric, fluorescent, and electrochemical analyses. In this section, optical and electrochemical aptasensors based on smartphones will be explored in detail.

#### 3.3.1. Smartphone-Based Optical Aptasensors

Optical aptasensors are capable of inducing changes in light absorption and luminescence upon interaction with various analytes, offering advantages such as cost-effectiveness, simple labeling, and low reagent requirements [89]. A myriad of smartphone-based optical aptasensors primarily leverage the high-resolution camera equipped in smartphones. Most optical-based analytical methodologies can be integrated with smartphones, utilizing cameras as ‘smart detector’, image sensors as ‘smart recorder’, and specialized APPs as ‘smart readout’ [93,94].

In particular, colorimetric detection garners significant attention due to its simplicity, where a smartphone alone can capture colored images via its camera [95]. Quantitative image analysis performed on a smartphone can interpret color profiles, particularly the red, green, and blue (RGB) values within the monitoring area. According to the grayscale or RGB value, the results can elucidate the concentration of the analyte [96].

**Figure 4:**
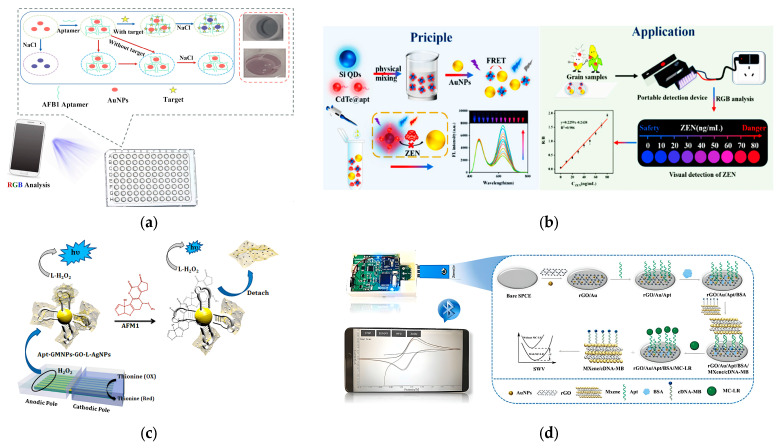
(**a**) Schematic diagram of the smartphone-based colorimetric aptasensor for AFB1 detection. Reprinted with permission from Ref. [98]. Copyright 2023, Elsevier. (**b**) Mechanism diagram of the smartphone-based fluorescent aptasensor for ZEN detection. Reprinted with permission from Ref. [102]. Copyright 2024, Elsevier. (**c**) Schematic representation of the amplified visual electrochemiluminescence detection of AFM1. Reprinted with permission from Ref. [103]. Copyright 2018, Elsevier. (**d**) Schematic diagram of portable electrochemical aptasensor for MC-LR detection. Reprinted with permission from Ref. [104]. Copyright 2022, Elsevier.

The utilization of GNPs in aptamer-based colorimetric detection for color signal generation has been widely demonstrated, resulting in discernible color changes observable with the naked eye [97]. For example, Lu et al. [98] reported a simple and intuitive smartphone-assisted colorimetric aptasensor, employing GNPs as indicators for measuring AFB1 (Figure 4a). In the absence of AFB1, the GNPs-aptamer conjugate remained stable even in high concentrations of NaCl, retaining the solution’s red color. Conversely, the presence of AFB1 led to the dissociation of the aptamer from the GNPs, with the aptamer specifically binding to AFB1, thereby inducing a color change to blue due to GNP aggregation. This color alteration could be easily captured with a smartphone, simplifying the detection of biotoxins, showing a good response range of 0.2–8.0 μg·kg^−1^ and a LOD of 0.08 μg·kg^−1^. Subsequently, Zheng et al. [99] engineered a novel colorimetric aptasensor that coupled the electrochemical reactions for the monitoring of fumonisin B1 (FB1) by using a smartphone. It was achieved by conjugating GNP-labeled aptamer on the surface for FB1 detection. Following the chemical reduction of Ag on GNPs, the Ag particles can catalyze H_2_O_2_ into oxygen bubbles. The bubble count significantly impacted the current flow, inhibiting the electro-deposition of Prussian blue at the indium tin oxide (ITO) driving electrode. With FB1, the GNPs-aptamer was dissociated because of the special binding between the aptamer and FB1, leading to a decreased number of Ag particles assembled on the glassy carbon electrode and an increment in Prussian blue at the ITO electrode. The current recovery, observable by the color change in Prussian blue with the naked eye, could be utilized to monitor the concentration of FB1 over the range of 0.001–10 μg·kg^−1^ through using a smartphone.

Fluorescent detection is a promising biosensing strategy with significant potential for in-field analysis, particularly when integrated with smartphones [100]. For the quick and simultaneous in-field analysis of biotoxins like ZEN and OTA, Lin et al. [101] devised a portable paper-based intelligent sensing system by combining a fluorescent LFA aptasensor with a smartphone spectrum reader. By using green and blue UCNPs as fluorescence labels functionalized with aptamer, the system swiftly captures fluorescence images with a portable smartphone upon the addition of ZEN and OTA. A color recognition app installed on the smartphone instantly analyzes the RGB values of the fluorescent images, enabling the detection of ZEN and OTA in food without the need for additional instruments, achieving LODs of 0.44 μg·kg^−1^ and 0.098 μg·kg^−1^ for the ZEN and OTA, showcasing, respectively, the linear ranges of 0.5–100 μg·kg^−1^ and 0.1–50 μg·kg^−1^. Furthermore, Song et al. [102] designed a fluorescence resonance energy transfer (FRET) ratiometric biosensor for ZEN detection using aptamer-modified CdTe quantum dots (QDs) and GNPs as the donor and acceptor, respectively (Figure 4b). The red fluorescence of CdTe QDs was quenched by GNPs through FRET, with Si QDs serving as an internal reference signal due to their inherent blue fluorescence. In the presence of ZEN, the aptamer-modified CdTe QDs bind to ZEN, increasing the acceptor–donor distance, thereby blocking FRET and restoring the fluorescence of CdTe QDs. The smartphone-based optical aptasensor was then constructed through the capture of fluorescence images and RGB value analysis. An LOD of 0.01387 μg·kg^−1^ was achieved based on the linear relationship with the concentration of ZEN and RGB values, which demonstrated visual POCT and on-site quantitative detection in the field.

Overall, superior advantages of smartphone-based optical aptasensors have caused the development of a detection range of biotoxins. Colorimetric detection, in particular, can appeal to a large number of concerns for commercialization due to its simplicity in obtaining preliminary experimental results. Utilizing a smartphone to directly observe color changes obviates the necessity for complex equipment, thereby rendering it a user-friendly approach. However, colorimetric detection still has limitations, such as complication sample preparation and relatively lower sensitivity, which may hinder its broader application. On the other hand, fluorescent detection faces the primary challenge of standardizing light sources. Despite numerous efforts to address this issue, the development of standard filters to block external light remains inadequate. This shortfall underscores a gap in the advancement of smartphone-based optical aptasensors for biotoxin detection. The current limitations highlight the necessity for ongoing development in this field to realize the full promise of smartphone-based optical aptasensors in biotoxin detection, thereby moving closer to practical, efficient, and user-friendly solutions for on-site biotoxin monitoring.

#### 3.3.2. Smartphone-Based Electrochemical Aptasensors

In recent times, smartphones have been connected to diverse kinds of electrochemical aptasensors, serving as tools to capture and conduct electrochemical signals [105]. Smartphone-based electrochemical aptasensors have emerged as exemplary methods for facile and rapid monitoring of analytes in food and environmental samples, owing to numerous dominances, such as lower sample quantity requirement, cost-effectiveness, heightened sensitivity, swift response, and time-efficient procedures, among others [106]. Typically, smartphones are paired with various electrochemical techniques like amperometric, voltammetric, and impedimetric methods for biotoxin monitoring [88]. For instance, Khoshfetrat et al. [103] introduced a sensitive electrochemical aptasensor for detecting aflatoxin M1 (AFM1) through utilizing a closed bipolar electrode (BPE) array. In this assay, aptamer-conjugated nanoparticle-coated magnetic Fe_3_O_4_ (Apt-GMNPs) were mixed with the Ag nanoparticle-decorated graphene oxide (GO-L-AgNPs). Upon introducing this complex to the BPE array, each individual electrode could detect a concentration of AFM1. The aptamer’s preferential interaction with the AFM1 led to the separation of GO-L-AgNPs, enabling the monitoring of H_2_O_2_ at the anodic poles and electrochemiluminescence (ECL) of luminol using ImageJ 1.4 software on a smartphone. This smartphone-based electrochemical aptasensor demonstrated a linear correlation between the concentration of AFM1 and the ECL image gray value within the range of 10 to 200 μg·kg^−1^, achieving a detection limit of 0.05 μg·kg^−1^ (Figure 4c). The control, reception, and visualization of the results were executed in real-time through Bluetooth and a mobile app on the smartphone (Figure 4d). Subsequently, Fan et al. [104] developed a smartphone-based portable electrochemical aptasensor by integrating a portable electrochemical system with square wave voltammetry (SWV), cyclic voltammetry (CV), and chronoamperometry (CA) modules, a screen-printed carbon electrode (SPCE), and a smartphone equipped with custom applications. The aptamer was anchored onto the SPCE, which was modified by GNPs and graphene oxide (rGO). The cDNA labeled with methylene blue was conjugated to MXene, forming a signal amplification probe. The target analyte (MC-LR) would compete with the probe for binding with the aptamer, culminating in a competitive aptasensor. The control, reception, and visualization of the results were executed in real-time through Bluetooth and a mobile APP on the smartphone for the detection of MC-LR, which demonstrated a linear range of 0.0001–5 μg·kg^−1^ and the LOD of 4 × 10^−5^ μg·kg^−1^ nM (Figure 4d).

In contrast to optical aptasensors, electrochemical aptasensors obviate the need for optical fibers or camera-based data collection systems and also circumvent the use of complex software for manipulation and data analysis. Despite the manifold advantages of associating electrochemical aptasensors with smartphones, scant instances are applied to biotoxins monitoring. Therefore, it is anticipated that the proliferation of these economical and portable electrochemical aptasensors will witness an uptick in the ensuing years.

### 3.4. Other Portable Aptasensors

Besides the afore-mentioned LFAs, PGMs and smartphones, researchers have explored the use of other devices for biotoxin detection, including pressure meters, thermometers, pH meters, pregnancy test strips and microfluidic chips. These endeavors aim to introduce novel biosensing methods and relevant products to the market.

#### 3.4.1. Pressure Meter-Based Aptasensors

**Figure 5:**
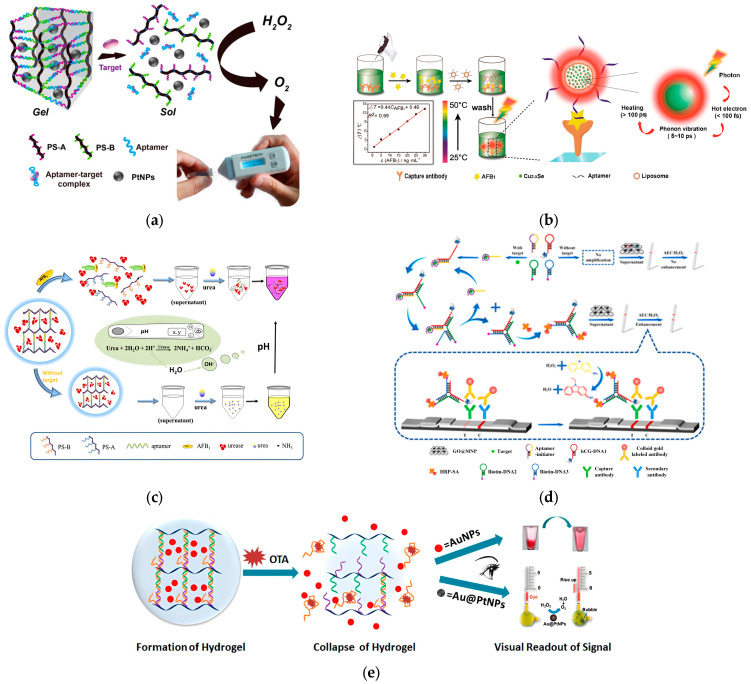
(**a**) Schematic illustration of a pressure meter-based aptasensor applied to OTA. Reprinted with permission from Ref. [107]. Copyright 2017, American Chemical Society. (**b**) Schematic protocol for AFB1 monitoring based on aptamer identification and a thermometer readout. Reprinted with permission from Ref. [109]. Copyright 2019, American Chemical Society. (**c**) Principle explanation of pH meter-based portable analysis of AFB1 coupled with an aptamer-crosslinked hydrogel. Reprinted with permission from Ref. [110]. Copyright 2018, Elsevier. (**d**) Overview of combining a catalytic hairpin assembly with pregnancy test strip for highly accurate monitoring of AFB1, OTA and ZEN. Reprinted with permission from Ref. [111]. Copyright 2022, Elsevier. (**e**) Working principle of DNA hydrogels with aptamer cross-links combining microfluidic chips for monitoring OTA. Reprinted with permission from Ref. [112]. Copyright 2015, American Chemical Society.

Recently, the pressure meter, serving as a novel transducer, has attracted intensive interest in the realm of portable biosensors. It translates the target recognition event into a pressure signal induced by gas generation. Within confined spaces, reactant molecules are converted into gas, engendering a significant pressure alteration and thereby discerning the target concentration. These pressure meter-based biosensors amplify signals and broaden the dynamic range. For instance, Liu et al. [107] reported a pressure meter-based aptasensor combined with a target-responsive hydrogel, paving the way for a simplistic, user-friendly, and sensitive detection of several toxic substances (Figure 5a). The hydrogel, encapsulating platinum nanoparticles (PtNPs), was cross-linked with aptamer strands through hybridization reactions. The target (OTA) could bind with aptamer, instigate the dissociation of DNA hydrogels, and consequently, release PtNPs for effective catalytic reactions. The liberated PtNPs could catalyze H_2_O_2_ to generate a substantial amount of oxygen (O_2_), resulting in a notable pressure change. Employing this strategy, the detection of OTA shows exceptional specificity and selectivity, spanning a range of 0 to 1.0 × 10^3^ μg·kg^−1^ with a detection limit of 15 μg·kg^−1^. Analogously, based on the DNA hydrogel-based method, Tang et al. [108] further discovered that when the target (AFB1) was present, the addition of exonuclease I (Exo I) could identify and excise the target–aptamer conjugate, facilitating the release of the AFB1 and aptamer. The released aptamer could re-interact with the hydrogel again, resulting in a target cycle for signal amplification. The proposed pressure meter-based aptasensor also demonstrated heightened sensitivity for AFB1 monitoring, with an LOD of 9.4 μg·kg^−1^.

#### 3.4.2. Thermometer-Based Aptasensors

Photothermal effects (PTEs) fundamentally revolve around the conversion of incident light into heat, showcasing immense potential in devising on-site monitoring methodologies. The only requisites for photothermal readout are a near-infrared laser and a portable thermometer. It is evident that utilizing a thermometer as a sensing readout tool bears numerous advantages, including low-cost, continuous enhancement in design and performance, and easy adaptability for home use, among others. Concurrently, the swift advancement of innovative and smart photothermal materials, such as gold nanomaterials, carbon nanomaterials, and semiconductor nanoparticles, has broadened the application spectrum.

Specifically, Li et al. [109] designed a unique thermometer-based aptasensor for the monitoring of AFB1 employing plasmonic copper selenide nanocrystals (Cu_2_-xSe NCs) as the photothermal conversion reagents (Figure 5b). Photothermal soft nanoballs were initially fabricated by encapsulating Cu_2_-xSe NCs into liposomes, which were subsequently modified with aptamers on the surface. A sandwich formation of target AFB1 was then constructed between the aptamer in photothermal soft nanoballs and a captured antibody. Upon near-infrared irradiation, the heat conducted from the photothermal soft nanoballs due to the photothermal conversion, elevated the temperature of the matrix solution. The rising temperature recorded by a portable thermometer exhibited a direct relationship with the AFB1 concentrations, enabling amplified detection in food with a LOD of 1 μg·kg^−1^. This thermometer-based aptasensor unveils a new avenue for real-time biotoxins detection. Motivated by the afore mentioned thermometer-based aptasensor, Lu et al. [113] further developed a thermometer-based nanozyme-linked aptasensor for AFB1 monitoring, utilizing Prussian blue nanoparticles (PBNPs) anchored to magnetic nanoparticles. It is exhilarating to note that the PBNPs rendered exceptional peroxidase-like activity, accompanied by a color change for colorimetric detection. Furthermore, magnetic nanoparticles acted as quenchers, diminishing fluorescence, thus making a compelling choice for constructing fluorescence detection probes. Similarly, the magnetic nanoparticles, modified with a specific aptamer, constructed a sandwich formation between the aptamer and an antibody targeting the AFB1. Upon the introduction of HCl and K_4_Fe(CN)_6_ to the solution, PBNPs were generated in magnetic nanoparticles, releasing the aptamer and photothermal conversion signal in near-infrared irradiation; the temperature change was measured by a readily available thermometer. It is noteworthy that the synthesized PBNPs also exhibited exceptional peroxidase-like activity, accompanied by a color change for colorimetric detection. Moreover, magnetic nanoparticles could act as quenchers for the fluorescent probe to reveal the fluorescent detection. Therefore, this creative innovation furnished the AFB1 with a multi-signal readout, showcasing remarkable analytical properties with a limit of detection of 0.54 × 10^−6^ μg·kg^−1^, up to 6333 and 28 times less than for photothermal and colorimetric analyses, respectively.

#### 3.4.3. pH Meter-Based Aptasensors

Recently, the change in pH value had been harnessed as a readout mechanism to construct portable and sensitive biosensors. Owing to its low cost, ease of operation, market availability, high sensitivity, and the capability to measure variations as minute as 0.001 units in pH, the pH meter emerges as a favorable candidate [114,115,116]. The genesis of acidic or alkaline substances through enzymatic reactions can be discerned using a pH meter. Among the catalytic enzymes utilized, urease and glucose oxidase are predominantly employed, with urease specifically being integrated with pH meter-based aptasensors for biotoxin monitoring. For example, Zhao et al. [110] pioneered a pH meter-based biosensor combined with aptamer-cross-linked hydrogel to monitor AFB1. With AFB1, the aptamer is selectively integrated with AFB1, disintegrating the hydrogel and the subsequent release of urease. The liberated urease then catalyzes urea, engendering an elevation in pH value (Figure 5c). The proposed portable biosensor allowed a successful, sensitive and rapid detection of AFB1 in the food samples and opened up a new approach for further utilization of pH meters. The fluctuation in pH value exhibited a dynamic correlation with the concentration of AFB1, boasting a LOD of 31 μg·kg^−1^. This proposed portable biosensor facilitated the successfully rapid detection of AFB1 in food samples, thereby unveiling a novel avenue for the extended utilization of pH meters in biosensing applications.

#### 3.4.4. Pregnancy Test Strip-Based Aptasensors

Currently, although commercial test strips for a variety of biotoxins are available on the market, they typically require a color coating on the test strips, which not only elevates the detection cost but also prolongs the preparation time. In contrast, the widely utilized pregnancy test strips present an alternative strategy for constructing portable and sensitive biosensors due to their affordability and accessibility. Inspired by this development, Zhong et al. [111] successfully harnessed a pregnancy test strip to devise a common protocol for monitoring a wide range of biotoxins. In this assay, the specific aptamers were combined with catalytic hairpin assembly (CHA), which further facilitated signal amplification through subsequent enzymatic reactions, thereby enhancing sensitivity (Figure 5d). This signal amplification strategy obviated the need for test strip assembly and the preparation or purification of aptamer/antibody. Human chorionic gonadotropin (hCG) was conjugated with a probe to attain biotoxins monitoring through the CHA system and signal amplification. Utilizing the pregnancy test strip, aptasensors based on CHA, AFB1, OTA, and ZEN could be discerned within a mere 15 min by naked eye, with the LODs being 0.020, 0.050, and 0.020 μg·kg^−1^, respectively. The empirical studies underscored that the strategy could be used to analyze biotoxins economically, conveniently, accurately, and sensitively. Furthermore, the versatility of different DNA sequence designs can expand the spectrum of monitoring analytes, thereby propelling a novel trajectory for the employment of pregnancy test strips in the realm of food and environmental detection.

#### 3.4.5. Microfluidic Chip-Based Aptasensors

Among the various portable aptasensors, microfluidic chips have emerged as ideal analytical tools for portable assays, which ahve gained significant popularity in recent years. Microfluidic chip technology integrates essential processes, such as sample preparation, reaction, separation, and detection, into a micron-sized chip, allowing for the automatic completion of the entire analysis. These chips are crucial for detecting cells, nucleic acids, proteins, and other small molecules, and have been widely adopted for portable, rapid detection due to their convenience, sensitivity, and low cost.

Recently, various types of target-responsive DNA hydrogels with aptamer cross-links have been developed for use in microfluidic chip-based portable aptasensors. DNA hydrogels intelligently collapsed upon target recognition, showing the advantages of specificity, accessibility, and affordability. Liu et al. [112] designed a microfluidic chip-based aptasensor using DNA hydrogel-entrapped AuNPs for the detection of OTA (Figure 5e). When OTA binds to the aptamer, hydrogel swells and releases AuNPs, which catalyze the decomposition of H_2_O_2_ to generate O_2_, producing a quantitative readout within the chip via pressure changes. Similarly, Ma et al. [117] employed a comparable approach, constructing an aptamer-crosslinked hydrogel entrapped with PtNPs for AFB1 detection. Integrated with a microfluidic chip, this method enabled the quantitative analysis of AFB1 concentrations in beer, achieving a detection limit of 0.553 μg·kg^−1^.

Electrochemical methods are also widely used in microfluidic chip-based aptasensors. These systems detect biotoxins within a microfluidic channel or chamber by forming an electrical signal through catalytic interactions with aptamers, with the signal correlating to the biotoxin concentration. Ramalingam et al. [118] developed an electrochemical microfluidic chip-based aptasensor for OA monitoring. This integrated device, composed of a polydimethylsiloxane microfluidic chip and a screen-printed carbon electrode (SPCE) modified with anti-OA aptamers, achieved an LOD of 0.002 μg·kg^−1^ for OA detection. This microfluidic electrochemical aptasensor is practical and accessible for on-site monitoring. Liu et al. [119] further enhanced microfluidic chip-based aptasensors by integrating electrochemiluminescence (ECL) technology, which is known for its excellent sensitivity and low background interference. They developed a portable visual microfluidic chip consisting of a sensing cell and a reporting cell. Upon detecting OTA, aptamers were released in the sensing cell, causing a decrease in the impedance of the electrode. Meanwhile, in the reporting cell, the ECL transmitter from Ru(bpy)_3_^2+^/bismuth oxyhalides (BiOI) microspheres was enhanced. Analyzing changes in the intensity of the ECL image makes it possible to establish the concentration of OTA without the need for specific devices. This visible ECL aptasensor chip is portable, cost-effective, and shows impressive performance in the detection of beer samples.

The microfluidics-aptasensor system coupled with magnetic separation was performed by He et al. [120] They designed three kinds of aptamers based tripartite DNA structure-functionalized Au-Fe_3_O_4_ magnetic nanocomposites for the simultaneous detection of aflatoxin M1, kanamycin and 17β-estradiol in milk. The strategy successfully facilitated the recognition between the aptasensors and analyte and enabled the straightforward separation by magnetic beads, thereby solving the complex matrix interference in real samples. Notably, analyte-induced tripartite DNA structural change generated rolling circle amplification (RCA), significantly increasing the sensitivity of the assay.

#### 3.4.6. Portable Potentiostat-Based Aptasensors

The continuous, real-time determination of biotoxins in complex samples is crucial for various sectors, including agriculture, food safety, and food production. Accordingly, Somerson et al. [121] described a portable potentiostat-based electrochemical aptasensor that supports continuous and real-time detection of OTA in a flowing stream of foodstuffs. This aptasensor is composed of an OTA-binding aptamer, which is attached to the gold electrode at its 5′ end via a six-carbon thiol linker and is oxidized with a methylene blue redox reporter at its 3′ end. OTA binding changes the conformation of the aptamer, which alters the rate of electron transfer easily detected by square wave voltammetry to quantify the concentration of the target observing a change in measured current. Such detections only depend on a simple, inexpensive portable potentiostat that achieves a continuous, real-time, rapid determination, fully responding to 3.23 × 10^5^ μg·kg^−1^ OTA within the few seconds. The portable potentiostat-based aptasensors for OTA detection directly in flowing foodstuffs without the addition of any modifying reagents enable the continuous determination of food processing workflows, thereby propelling a novel, fast, simple assurance against food taint.

## 4. Discussion

The integration of advancements in aptasensing technology with the enhanced features of portable devices, including LFAs, PGMs, smartphones, pH meters, pressure meters, and thermometers, has catalyzed the development of portable aptasensors. As highlighted in the preceding literature, these portable aptasensors exhibit high sensitivity and selectivity in monitoring biotoxins in real samples, allowing non-professionals to conduct analyses anytime, anywhere. While these sensing strategies offer numerous advantages, such as reliable quantitative results, cost-effectiveness, compactness, and rapid analysis, there are still challenges and limitations to overcome for expanding their applications.

(1) Aptamers, as molecular recognition elements, are significantly more cost-effective and efficient than antibodies. However, despite the identification of over two thousand biotoxins species globally [122], the application of aptamers in portable device-based aptasensors for biotoxin detection remains limited. This underscores the imperative for the selection of more diverse and effective aptamers to broaden the scope of applications.
(2) In many cases, the sensitivity of portable aptasensors is insufficient for detecting biotoxins in complex food or environmental matrices. To realize portable and sensitive applications, integrating signal amplification strategies into these aptasensors is crucial. For example, developing portable aptasensors for biotoxins with amplification techniques such as hybridization chain reaction (HCR), rolling circle amplification (RCA), polymerase chain reaction (PCR), and recombinase polymerase amplification (RPA) presents a promising approach. Additionally, applying advanced nanomaterials can enhance the quantification of ultra-low levels of biotoxins. For example, some crystalline porous nanomaterials have garnered substantial research attention, including hydrogel [123] and metal–organic framework (MOF) [124], due to their promising potential in the development of nanomaterials as nanocarriers for signal amplification.
(3) Continuous monitoring through biosensors is crucial for many sectors, including agriculture and food safety. Achieving continuous biotoxin monitoring is essential for ensuring food safety, understanding contamination patterns, evaluating control measures, and safeguarding public health. Traditional real-time monitoring has primarily focused on metabolites, nutrition-related peptide hormones, and protein biomarkers, as fluctuations in their systemic concentrations provide critical information for guiding real-time clinical interventions [125,126,127,128]. In contrast, real-time detection of biotoxins is a relatively new concept, but it holds significant potential for improving biotoxin monitoring [129]. While aptamer-based biosensors are rarely used for continuous biotoxin monitoring, developing portable aptasensors with POCT capabilities for this purpose is highly desirable. For a portable aptasensor to be truly useful for continuous monitoring, the device must be sufficiently selective to detect biotoxins directly in unfiltered, unadulterated media, and it must exhibit high specificity for the targets.
(4) The functionalization of aptamers onto DNA nanostructures, or origami, has emerged as a powerful approach for developing advanced aptasensors. By providing a stable and rigid platform for aptamer immobilization, DNA nanostructures can enhance the stability, sensitivity, and specificity of these sensors. The precise control over the spatial arrangement of aptamers on the DNA nanostructure allows for the design of multiplexed aptasensors and minimizes crosstalk between different aptamers [130,131]. Moreover, DNA nanostructures can be engineered to incorporate additional functional elements, such as fluorophores or electrochemical tags, enabling signal amplification and further enhancing sensor sensitivity [132]. Numerous instances of aptasensors utilizing DNA nanostructures have been documented [133,134,135]. The unique structure of tetrahedral DNA nanostructures (TDNs) allows for the efficient and rapid binding of target molecules to the electrode surface, resulting in amplified electrochemical signals [136,137]. Additionally, the incorporation of a DNA walker on DNA nanostructures can further improve detection sensitivity, eliminating the high cost and instability associated with enzyme-assisted amplification techniques [138,139]. Nevertheless, the majority of DNA nanostructure-based aptasensors continue to depend on fluorescence or electrochemical workstations for measurement. The functionalization of aptamers onto DNA nanostructures, or origami, constitutes a substantial advancement in the development of aptamer-based sensors. The integration of this methodology with portable devices for the detection of biotoxins is highly desirable.
(5) The market offers a wide array of portable devices, each with its own features, which must be carefully considered. Furthermore, the absence of standardization in this field complicates the comparison of monitoring results across different studies. Standardization is pivotal for the development and validation of portable aptasensors intended for food and environmental safety and hazard analysis. It is also crucial to validate the reliability and accuracy of these aptasensors against established devices and standard approaches.
(6) Most reported portable aptasensors are designed for single biotoxin detection, with only a few capable of multi-analyte detection. Given the coexistence of various biotoxins in food or environmental matrices, it is imperative to develop more portable aptasensors capable of simultaneously monitoring multiple biotoxins in real-world samples. Addressing this challenge should be a priority for future research.
(7) Currently, portable device-based aptasensors have not yet reached commercial availability, and the associated technologies and methodologies are still in the early stages of development. We believe that an electrochemical portable device could represent the ultimate form of aptasensors for biotoxin detection, similar to integrated redox-electro-reactor glucometers. While aptasensors have been widely applied in the field of electrochemistry [140,141,142], it is unfortunate that most current research still relies on electrochemical workstations for detection. The integration of aptasensors with electrochemical principles to develop fully functional portable devices capable of quantitative detection remains a significant challenge.

## 5. Conclusions

The burgeoning risks associated with biotoxins pose severe threats to human health, underscoring the critical need for sensitive and reliable strategies for the detection of biotoxins in environmental and food matrices. Conventional analytical methodologies such as HPLC/HPLC-MS and ELISA often face limitations due to high operational costs, the requirement for specialized expertise, and complicated protocols. Over the past decade, substantial advancements in portable aptasensors have emerged, presenting a promising alternative due to their compactness, cost-effectiveness, user-friendliness, and real-time monitoring capabilities. In this review, we have systematically examined and summarized recent advancements in portable aptasensors for biotoxin detection. The employment of aptamers as recognition elements presents a promising approach for target identification in complex matrices, with signal generation facilitated through devices such as LFAs, PGMs, smartphones, pH meters, pressure meters, and thermometers. We have highlighted key advancements in the field and the potential of these technologies. Future efforts should focus on expanding the application of portable aptasensors, overcoming existing challenges, and facilitating the transition of these automatic and integrated devices to the market. Given their widespread accessibility and significant potential, portable aptasensors represent a new paradigm for biotoxin detection in food safety and environmental monitoring.

As concluded, the integration of portable aptasensors with diverse signal amplification strategies is gradually gaining momentum in biotoxin monitoring, driving considerable advancements in the field. The incorporation of advanced nanomaterials such as GNPs, PtNPs, AgNPs, and MOFs into portable aptasensors is poised to further enhance analytical performance. There remains a necessity for continued exploration and investigation of more nanomaterials that can be utilized in portable aptasensors. Moreover, the adoption of novel nucleic acid amplifications, such as RCA, HCR, PCR, RPA, and clustered regularly interspaced short palindromic repeats (CRISPR), is anticipated to augment the sensitivity of the assays.

Furthermore, more attention should be focused on the simplification of the user interface of these devices to really qualify them as true point-of-care testing (POCT) tools for biotoxins. The ideal interface would involve a straightforward process where the sample is combined with a reagent in a single step, followed by signal detection. This approach envisions encapsulating the complexity within the devices and reagents themselves—a strategy that, to our knowledge, has yet to be fully explored in the literature. Future directions should transcend laboratory research and move toward the commercialization of portable aptasensors, thereby fully integrating these biosensors into a simple, real-time, in situ, and user-friendly format. It is envisaged that, through the collaborative efforts of researchers, novel portable aptasensors that are both cost-effective and rapid will be conceived and widely deployed in the field of biotoxin detection.

## Figures and Tables

**Table 2:** Comparison of aptamers and antibodies as bioreceptors.

Features	Aptamer 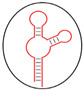	Antibody 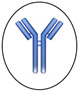
Ingredient	Oligonucleotides (DNA or RNA)	Proteins
Target	Small molecules, proteins, cells, bacteria, viruses and parasites	Proteins and peptides
Size	Small (10~20 kDa)	Large (150 kDa)
Synthesis method	Chemical synthesis	Biological manufacturing
Immunogenicity	Low	High
Affinity	High (10 pM to 10 μM)	High (10 pM to 10 μM)
Specificity	High	High
Stability	High (stable at room temperature)	Low (Refrigerated for storage)
Development time	2~6 weeks	6~18 months
Chemical modifications	Simple and controllable	Restricted and uncontrolled
Cost	Low	High

**Table 3:** The summary of the aptamers and aptasensors developed against biotoxins.

Target	Aptamer Sequence (5′–3′)	Secondary Structure	Selection Method	Aptasensor Type	Linear Range μg·kg^−1^	LOD μg·kg^−1^	Ref.
CTX-MI	TTTGGGGATGG GCAACG GTAAAAAGGGTCAAAAGGCTTTT	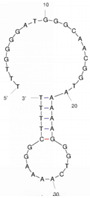	Mag-SELEX	BLI aptasensor	1.5 × 10^3^~7.5 × 10^4^	390	[40]
CT	GGCAAAAAGGATTGCCCAGGTCTGCTGTCTAGCC GGATTC	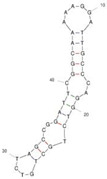	Mag-SELEX	Enzyme-linked aptamer assay	1~1000	2.1	[41]
GTX	AACCTTTGGTCGG GCAAGGTAGGTT	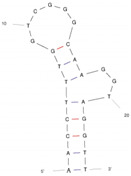	GO-SELEX	Label-free and real-time optical BLI aptasensor	0.2~90	0.050	[52]
GYM-A	GCGA CCGAAGTGAGGCTCGATCCAAGG	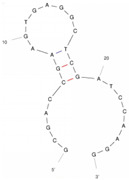	Capture-SELEX	BLI aptasensor	28~444	3.15	[53]
STX	GGCGGGTTT TGAGGGTC GCATCCCGT GGAAACAGGTTCATTGTTCCCGCC	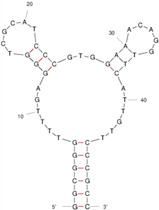	Mag-SELEX	Label-free colorimetric aptasensor	0.04356~11.15	0.04255	[42]
AFB1	GTTGGGCA CGTGT TGTCTCTCTGTGTCTCGTGCCCTTCGCTAGGCCCACA	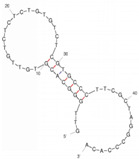	Mag-SELEX	Colorimetric aptasensor	1.56~1599	0.587	[43]
Patulin (PAT)	GGCCCGCCAACCCGCATCATCTACACTGATATTTTACCTT	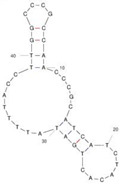	GO-SELEX	Colorimetric aptasensor	0.050~2.50	0.048	[54]
Trichothecene mycotoxin (T-2 Toxin)	GTATATCAAGCATCGCGTGTTTACACATGCG AGAGGTGAA	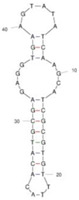	GO-SELEX	Fluorescent aptasensor	233~ 1.75 × 10^4^	186	[55]
PTX	GGAGGTGGTGGG GACTTTGCTTGTA CTGGGCGCCCGG TTGAA	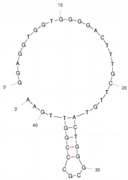	Mag-SELEX	Real-time optical BLI aptasensor	0.20~ 0.70	0.04 × 10^−3^	[44]
20 Methyl Spirolide Gb (SPX G)	CACGACGAGCG ATAGGTTGT GGACATTGACA GGACCGAACA CGCGCCCC	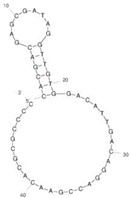	Bead SELEX	Microscale thermophoresis aptasensor	0.0019~ 125	0.39 × 10^−3^	[56]
OA	GGTCACCAAC AACAGGGAGC GCTACGCGAA GGGTCAATGT GACGTCATGC GGATGTGTGG	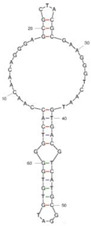	Bead SELEX	Label-free electrochemical aptasensor	0.1~60	0.070	[57]
Microcystin-leucine arginine (MC-LR)	GGCGCCAAA CAGGACCA CCATGACAATTA CCCATACCAC CTCATTATGCC CCATCTCCGC	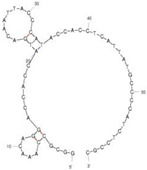	Bead SELEX	Electrochemical aptasensor	0.0075~ 0.0127	0.010	[58]
ZEN	ATACCAGCTT ATTCAATTCT ACCAGCTTT GAGGCTCGATCC AGCTTATTCAATTATACCAGCTTA TTCAATTATA CCAGCACAATC GTAATCAGTTAG	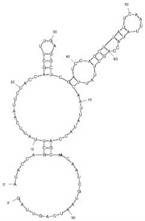	Capture-SELEX	Lable-free aptasensor	3.98~128	3.98	[59]
Ricin	ACACCCACCGCAGGCAGACGCAACGCCTCGGAGACTAGCC	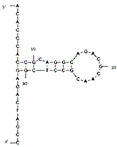	Bead SELEX	surface enhanced Raman scattering (SERS) aptasensor	0~50	25	[60]
Botulinum toxin	AGGGAAAATTTGACACTTTTCAAAC TGTCCTATGACA GTCCATAGG	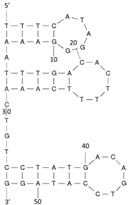	Mag-SELEX	Electrochemical aptasensor	15~1500	300	[61]
Staphylococcal enterotoxins	AGCAGCACAGAGGTCAGATGTACTTATGCATTTCCTCCCACGATCTTATTTGAGAGTGACCCTATGCGTGCTACCGTGAA	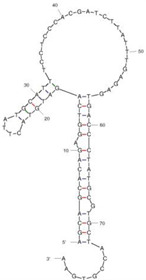	Mag-SELEX	Fluorescent aptasensor	0~1.0 × 10^4^	8.7	[62]

## Data Availability

Not applicable.
